# Perineural invasion/lymphovascular invasion double positive predicts distant metastasis and poor survival in T3–4 oral squamous cell carcinoma

**DOI:** 10.1038/s41598-021-99280-2

**Published:** 2021-10-05

**Authors:** Kuan-Chung Ting, Tsung-Lun Lee, Wing-Yin Li, Chia-Fan Chang, Pen-Yuan Chu, Yi-Fen Wang, Shyh-Kuan Tai

**Affiliations:** 1 Department of Otolaryngology-Head and Neck Surgery, Taipei Veterans General Hospital, No 201, Sec 2, Shipai Rd, Taipei, 11217 Taiwan; 2 Department of Otolaryngology, National Yang Ming Chiao Tung University, Taipei, Taiwan; 3 Department of Pathology, Taipei Veterans General Hospital, Taipei, Taiwan; 4 Institute of Clinical Medicine, National Yang Ming Chiao Tung University, Taipei, Taiwan

**Keywords:** Diseases, Oncology

## Abstract

Postoperative adjuvant therapy has been indicated by advanced T classification for T3–4 oral squamous cell carcinoma (OSCC) and the significance of perineural invasion (PNI) and lymphovascular invasion (LVI) in treatment for T3–4 OSCC remains unclear. Ninety-eight cumulative patients with T3–4 OSCC who underwent curative surgery between Jan 2002 and Dec 2010 were recruited and analyzed. Twenty-seven (27.6%) patients were PNI/LVI double positive. PNI/LVI double positive demonstrated independent predictive values for higher neck metastasis (LN+), higher distant metastasis (DM) and low 5-year disease-specific survival (DSS) rates (*p* < 0.001, *p* = 0.017, and *p* < 0.001, respectively) after controlling for other pathologic features of the primary tumors. A high DM rate of 33.3% was noted in PNI/LVI double-positive patients. Among the PNI/LVI double negative, single positive to double positive subgroups, increasing LN+, DM rates and decreasing DSS rate were observed. Among the 44 LN+ patients, PNI/LVI double positive remained associated with a markedly high DM rate of 42.9% and a poor 5-year DSS of 27.7%. PNI/LVI double positive plays important roles in prognostication and potential clinical application for T3–4 OSCC by independently predicting LN+, DM, and poor DSS, and can be used as a good marker to select DM high-risk patients for novel adjuvant therapy trials.

## Introduction

Oral squamous cell carcinoma (OSCC) is the most common type of head and neck cancer^1^ and the fourth common male cancer death in Taiwan. Surgery is the mainstay of treatment for OSCC, and pathologic features are commonly used for risk group stratification and treatment decision. Perineural invasion (PNI) and lymphovascular invasion (LVI) are two pathologic features representing the consequence of tumor microenvironment interactions. They are established poor prognostic factors in many human malignancies^2–6^. In OSCC, either PNI or LVI has been shown to correlate with advanced tumor stage, neck metastasis and poor survival^6–10^, and are two required elements in standard pathology report for OSCC^11^. In the National Comprehensive Cancer Network guidelines, both PNI and LVI are adverse pathologic features indicating the need for postoperative adjuvant therapy^12^.

T1–2 OSCC can be treated by single modality of surgery, and adjuvant therapy is generally not indicated in the absence of neck metastasis or adverse pathologic features^13–15^. Compared with T1–2 OSCC, T3–4 OSCC generally has a higher neck metastasis rate and poor prognosis, and aggressive combination treatment of surgery and postoperative radiotherapy (PORT) or chemoradiotherapy (POCCRT) is frequently required^16,17^. The advanced T classification has already been regarded as one adverse feature for treatment decision in treatment guidelines^12^, although pathologic features, such as PNI and LVI, appear at a much higher frequency in T3–4 OSCC^18,19^. Thus, the significance and clinical application of PNI and LVI in the treatment decision for T3–4 OSCC remain unclear.

Most studies of PNI and LVI in OSCC included patients of all T classifications^6–10^. Many recent studies, including ours, have focused on the role of PNI or LVI in early T1–2 OSCC^20–24^, but few studies have focused on T3–4 OSCC which has more aggressive clinical courses and poor prognosis. This retrospective study aimed to investigate the role of pathologic features of primary tumor, particularly PNI and LVI, in T3–4 OSCC and explore their potential clinical applications.

## Results

### Patient characteristics

Of the 98 T3–4 OSCC patients, 93 were male and 5 were female, with a mean age of 54 years (range 32–91 years). The clinicopathologic features are summarized in Table 1. The buccal mucosa (34.7%) and tongue (29.6%) were the two most common primary subsites. Twenty-eight (28.6%) patients were classified as pT3 and 70 (71.4%) as pT4. Positive margin was noted in 20 (20.4%) patients. With a mean tumor thickness 14 mm as the cutoff, 60 (61.2%) patients were classified as having thick tumors (> 14 mm). PNI was identified in 44 (44.9%) patients, and the same incidence of 44.9% was also noted for LVI. Fifty-five (56.1%) patients received postoperative adjuvant therapy. The mean follow-up time for surviving patients is 54.9 months (range 8–166 months).

**Table 1:** Clinicopathologic features of the 98 patients with T3–4 oral squamous cell carcinoma.

Variables	No. (%)	5-year DSS rate (%)	p*
**Subsite**			0.603
Buccal	34 (34.7)	75.2	
Tongue	29 (29.6)	61.8	
Gum	19 (19.4)	72.1	
Hard palate	8 (8.2)	66.7	
Others	8 (8.1)	80.0	
**pT classification**			0.325
T3	28 (28.6)	79.6	
T4	70 (71.4)	67.2	
**pN classification**			< 0.001
N0	54 (55.1)	88.5	
N1	6 (6.1)	60.0	
N2	38 (38.8)	47.6	
**Resection margin**			0.064
Non-positive	78 (79.6)	75.1	
Positive	20 (20.4)	54.1	
**Tumor thickness**			0.044
0–14 mm	38 (38.8)	83.8	
> 14 mm	60 (61.2)	63.2	
**Differentiation**			0.489
Well	77 (78.6)	71.5	
Moderate to poor	21 (21.4)	68.8	
**PNI**			0.002
No	54 (55.1)	82.8	
Yes	44 (44.9)	54.5	
**LVI**			0.005
No	54 (55.1)	81.9	
Yes	44 (44.9)	57.5	
**ENE**			0.001
Negative	77 (78.6)	77.0	
Positive	21 (21.4)	48.1	
**Adjuvant therapy**			0.015
No	43 (43.9)	83.4	
Yes	55 (56.1)	60.5	
PORT	21 (21.4)	82.9	
POCCRT	34 (34.7)	47.8	

*PNI* perineural invasion, *LVI* lymphovascular invasion, *ENE* extranodal extension, *PORT* postoperative radiotherapy, *POCCRT* postoperative concurrent chemoradiotherapy.

*Log-rank test.

### Predictors for neck metastasis and oncologic controls

Of the 98 T3–4 OSCC patients, neck metastasis (LN+) was shown in 44 (44.9%). We found that LVI (*p* < 0.001) and differentiation (*p* = 0.002) independently predicted LN+ after controlling for PNI, T classification and tumor thickness in multivariate analysis (Table 2). Local recurrence and regional recurrence occurred in 18 (18.4%) and 10 (10.2%) patients, respectively, but no pathologic feature predicted both events in our cohort. Distant metastasis (DM) developed in 17 (17.3%) patients, most commonly in the lung, and 9 (52.9%) occurred as isolated DM without local or regional recurrence. LN+ was found associated with the development of DM (31.8% vs. 5.6%; *p* = 0.001). Notably, POCCRT was done at a higher percentage among patients with DM than those without DM (82.4% vs 51.3%, *p* = 0.017). Trends of higher local recurrence (35.3% vs 14.8%, *p* = 0.057) and regional recurrence (23.5% vs 7.4%, *p* = 0.068) rates are also observed among patients with DM. The mean time of DM development was 9.0 months (range 3–24 months), and the mean survival time after DM diagnosis was 3.76 months (range 0–11 months). The 5-year disease-specific survival (DSS) and overall survival (OS) were 70.6% and 62.5%, respectively.

**Table 2:** Multivariate analyses of independent predictors of primary OSCC for LN+, DM and DSS (PNI and LVI separated).

Variable	*p* value	HR	95% CI
LN+*			
PNI (positive vs. negative)	0.091	2.38	0.87–6.54
LVI (positive vs. negative)	< 0.001	8.33	2.92–23.79
pT classification (T4 vs. T3)	0.888	1.08	0.36–3.23
Tumor thickness (> 14 mm vs. ≤ 14 mm)	0.702	1.22	0.44–3.42
Differentiation (moderate to poor vs. well)	0.002	7.99	2.14–29.78
DM*			
PNI (positive vs. negative)	0.172	2.33	0.69–7.87
LVI (positive vs. negative)	0.155	2.47	0.71–8.57
pT classification (T4 vs. T3)	0.551	1.55	0.37–6.45
Tumor thickness (> 14 mm vs. ≤ 14 mm)	0.026	11.09	1.34–91.74
Differentiation (moderate to poor vs. well)	0.239	2.41	0.56–10.38
Resection margin (positive vs. non-positive)	0.118	2.98	0.76–11.70
DSS**			
PNI (positive vs. negative)	0.010	3.16	1.32–7.54
LVI (positive vs. negative)	0.049	2.48	1.00–6.14
pT classification (T4 vs. T3)	0.297	1.70	0.63–4.57
Tumor thickness (> 14 mm vs. ≤ 14 mm)	0.203	1.91	0.71–5.16
Differentiation (moderate to poor vs. well)	0.074	2.51	0.91–6.91
Resection margin (positive vs. non-positive)	0.079	2.18	0.91–5.22

*OSCC* oral squamous cell carcinoma, *PNI* perineural invasion, *LVI* lymphovascular invasion, *LN*+ neck metastasis, *DM* distant metastasis, *DSS* disease-specific survival.

*Binary logistic regression.

**Cox proportional hazards model.

Preliminary multivariate analyses were performed for the independent roles of pathologic features of the primary tumors (Table 2). We found that only tumor thickness independently predicted DM (*p* = 0.026) after controlling for PNI, LVI, T classification, differentiation, and margin. Independent predictors for DSS were PNI (*p* = 0.010) and LVI (*p* = 0.049) after controlling for T classification, thickness, differentiation, and margin. In summary, when PNI and LVI were separately considered, PNI predicted a worse DSS, LVI predicted LN+ and a worse DSS, and neither PNI nor LVI predicted DM independently.

### Significance of PNI/LVI double positive

**Figure 1:**
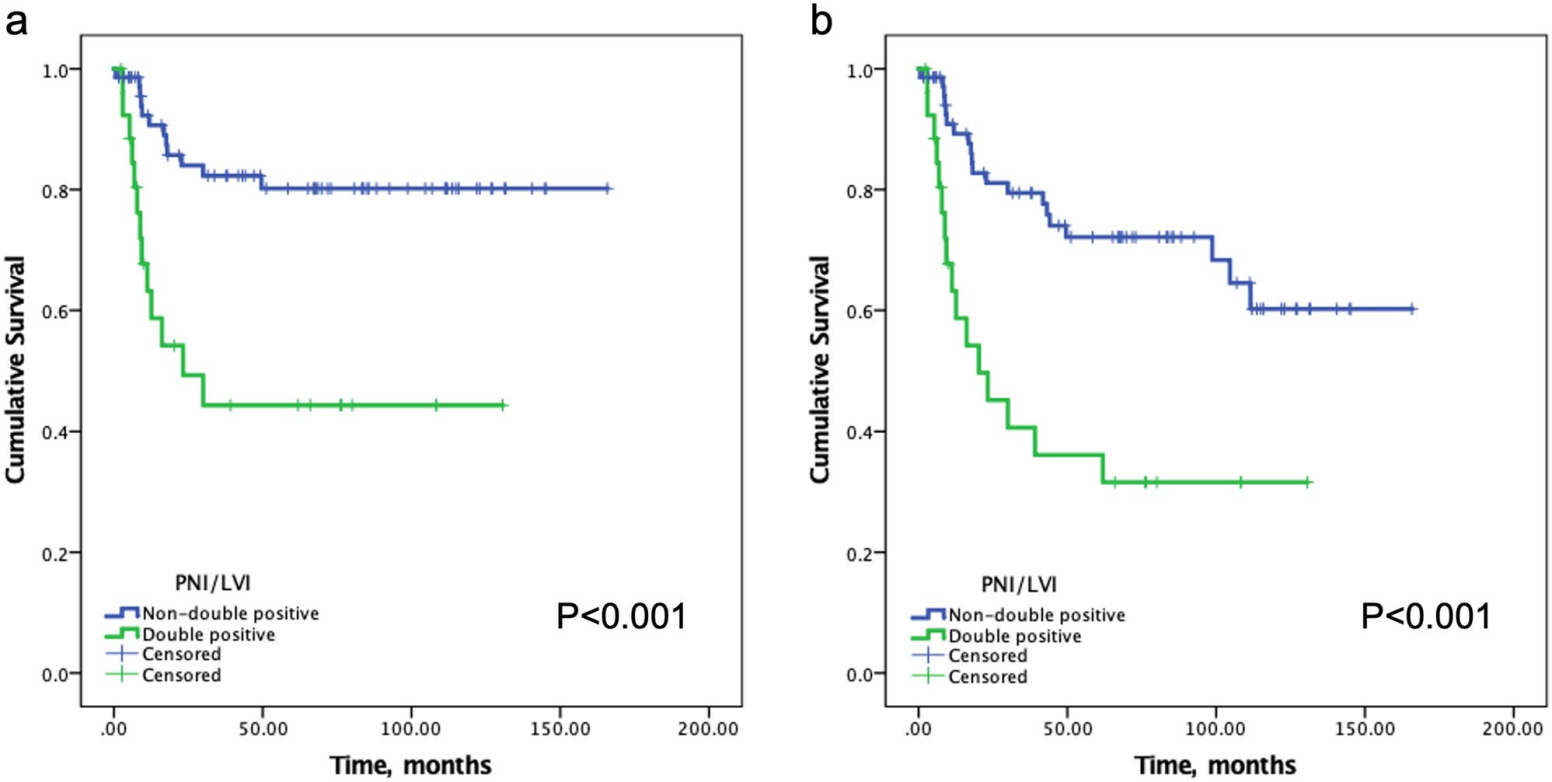
(**a**) Disease-specific survival curves for PNI/LVI double-positive and non-double-positive groups. (**b**) Overall survival curves for PNI/LVI double-positive and non-double-positive group.

Given the inconsistent predictive values of PNI and LVI for LN+, DM and DSS, further analyses were performed by combining PNI and LVI as one pathologic feature. Twenty-seven (27.6%) patients with both PNI and LVI were classified as PNI/LVI double positive. The other 71 (72.4%) patients were classified as non-double positive, including 34 (34.7%) as single positive (either PNI or LVI) and 37 (37.8%) as double negative. Univariate analyses demonstrated significant and higher impacts by PNI/LVI double positive on LN+ (77.8%), DM (33.3%) and 5-year DSS (44.3%) rates, compared with the impacts by either PNI or LVI (Table 3 and Fig. 1). Importantly, as shown in Table 4, multivariate analyses demonstrated that PNI/LVI double positive independently predicted significantly worse outcomes for LN+ (*p* < 0.001), DM (*p* = 0.017) and DSS (*p* < 0.001) after controlling for other pathologic features of the primary tumors.

**Table 3:** Univariate analysis of neck metastasis, distant metastasis, and 5-year DSS.

Variables	LN+*		Distant metastasis*		5-year DSS**	
	No. (%)	*p*	No. (%)	*p*	%	*p*
**PNI**		0.011		0.071		0.002
No	18/54 (33.3)		6/54 (11.1)		82.8	
Yes	26/44 (59.1)		11/44 (25.0)		54.5	
**LVI**		< 0.001		0.019		0.005
No	13/54 (24.1)		5/54 (9.3)		81.9	
Yes	31/44 (70.5)		12/44 (27.3)		57.5	
**PNI/LVI**		< 0.001		0.016		< 0.001
Non-double positive***	23/71 (32.4)		8/71 (11.3)		80.2	
Double positive	21/27 (77.8)		9/27 (33.3)		44.3	

*LN*+ neck metastasis, *DSS* disease-specific survival, *PNI* perineural invasion, *LVI* lymphovascular invasion.

*Pearson’s chi-square test or Fisher’s exact test.

**Log-rank test.

***Double negative or single positive.

**Table 4:** Multivariate analyses of independent predictors of primary OSCC for LN+, DM and DSS (PNI and LVI combined).

Variable	*p*	HR	95% CI
LN+*			
PNI/LVI double positive (double vs. not double)	< 0.001	9.44	3.12–28.59
pT classification (T4 vs. T3)	0.993	1.01	0.35–2.89
Tumor thickness (> 14 mm vs. ≤ 14 mm)	0.204	1.87	0.71–4.94
Differentiation (moderate to poor vs. well)	0.004	5.71	1.75–18.59
DM*			
PNI/LVI double positive (double vs. not double)	0.017	4.48	1.32–15.28
pT classification (T4 vs. T3)	0.618	1.45	0.34–6.30
Tumor thickness (> 14 mm vs. ≤ 14 mm)	0.015	14.07	1.67–118.76
Differentiation (moderate to poor vs. well)	0.259	2.37	0.53–10.54
Resection margin (positive vs. non-positive)	0.086	3.37	0.84–13.46
DSS**			
PNI/LVI double positive (double vs. not double)	< 0.001	4.44	1.92–10.26
pT classification (T4 vs. T3)	0.320	1.66	0.61–4.47
Tumor thickness (> 14 mm vs. ≤ 14 mm)	0.094	2.33	0.87–6.27
Differentiation (moderate to poor vs. well)	0.092	2.37	0.87–6.46
Resection margin (positive vs. non-positive)	0.068	2.22	0.94–5.25

*OSCC* oral squamous cell carcinoma, *PNI* perineural invasion, *LVI* lymphovascular invasion, *LN*+ neck metastasis, *DM* distant metastasis, *DSS* disease-specific survival.

*Binary logistic regression.

**Cox proportional hazards model.

**Figure 2:**
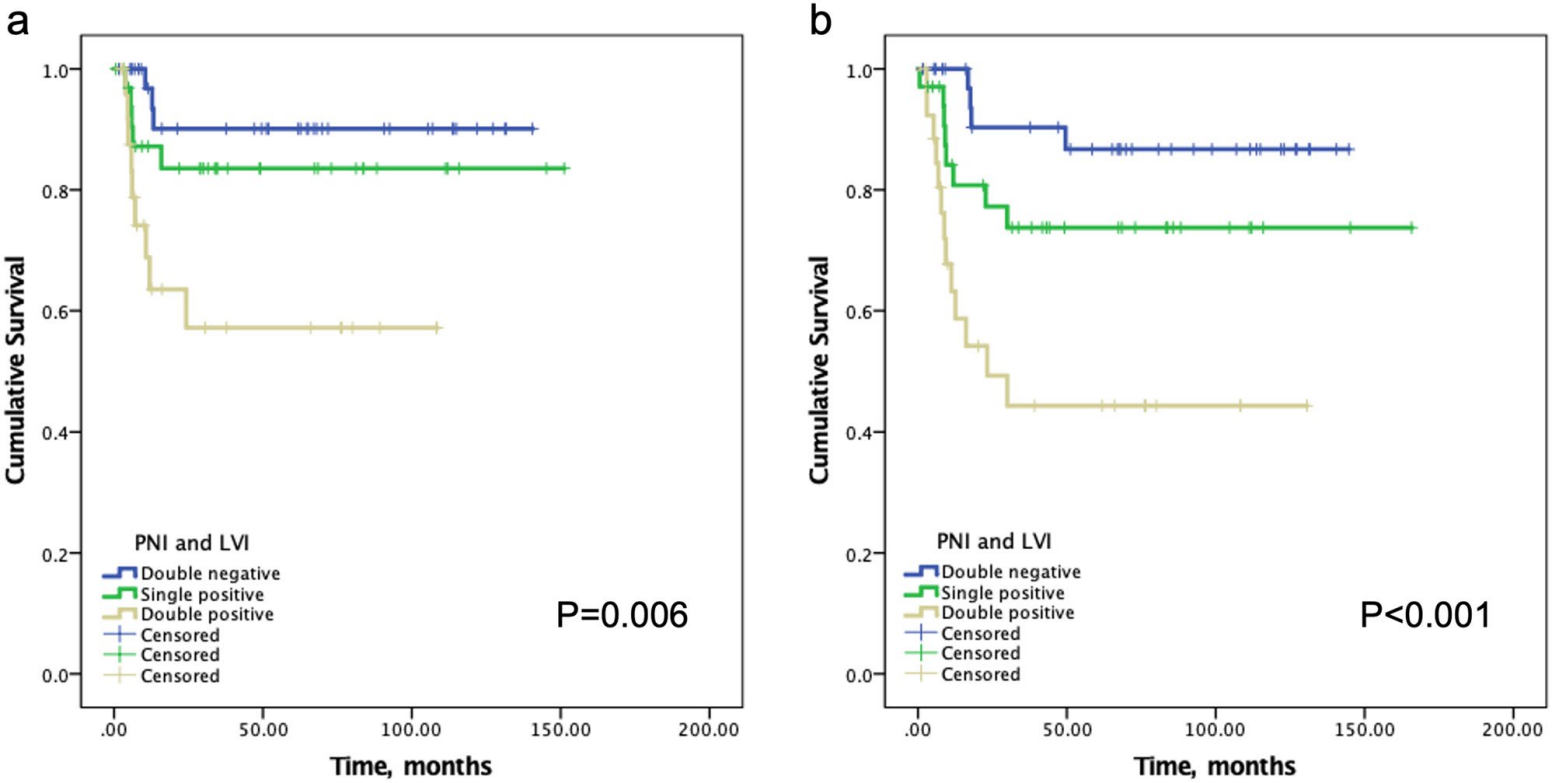
(**a**) Kaplan–Meier curves of distant metastasis (DM)-free survival according to PNI/LVI status. The 2-year DM-free survival rates were 90.1%, 83.6%, and 57.2% in PNI/LVI double-negative, single-positive and double-positive group, respectively. (**b**) Kaplan–Meier curves of disease-specific survival according to PNI/LVI status.

Using PNI/LVI double negative as the reference level, increasing LN+, DM and decreasing DSS rates were observed from the PNI/LVI double negative, single positive to double positive groups (Table 5). The DM-free and DSS survival rates among the three groups were significantly different (*p* = 0.006 and *p* < 0.001, respectively), particularly worse for the PNI/LVI double positive group (Fig. 2). Notably, in the 44 LN+ patients, the increasing impacts by combined PNI/LVI status remained consistent (Table 6). Increasing DM rates were observed from PNI/LVI double negative, single positive to double positive groups (12.5%, 26.7%, and 42.9%, respectively), and decreasing 5-year DSS rates were also clearly observed (83.3%, 59.3% and 27.7%, respectively).

**Table 5:** LN+, DM and 5-year DSS according to combined PNI and LVI status.

PNI/LVI status (n)	No (%) or rate	HR	95% CI	*p*
LN+*				
Double negative (37)	8 (21.6)	1		
Single positive (34)	15 (44.1)	2.86	1.02–8.06	0.046
Double positive (27)	21 (77.8)	12.69	3.83–42.05	< 0.001
DM*				
Double negative (37)	3 (8.1)	1		
Single positive (34)	5 (14.7)	1.95	0.43–8.89	0.386
Double positive (27)	9 (33.3)	5.67	1.36–23.59	0.017
5-year DSS**				
Double negative (37)	86.7%	1		
Single positive (34)	73.7%	2.43	0.73–8.07	0.148
Double positive (27)	44.3%	6.63	2.15–20.40	0.001

*PNI* perineural invasion, *LVI* lymphovascular invasion, *LN*+ neck metastasis, *DM* distant metastasis, *DSS* disease-specific survival.

*Binary logistic regression.

**Cox proportional hazards model.

**Table 6:** DM and 5-year DSS to combines PNI and LVI status in 44 LN+ patients.

PNI/LVI status (n)	No (%) or rate	HR	95% CI	*p*
**DM**				
Double negative (8)	1 (12.5)	1		
Single positive (15)	4 (26.7)	2.55	0.23–27.71	0.443
Double positive (21)	9 (42.9)	5.25	0.54–50.64	0.152
**5-year DSS**				
Double negative (8)	83.3%	1		
Single positive (15)	59.3%	3.60	0.43–29.88	0.236
Double positive (21)	27.7%	7.43	0.97–57.01	0.054

*PNI* perineural invasion, *LVI* lymphovascular invasion, *LN*+ neck metastasis, *DM* distant metastasis, *DSS* disease-specific survival.

## Discussion

Compared with T1–2 OSCC, patients with T3–4 OSCC are usually treated more aggressively based on the advanced T classification. Depth of invasion (DOI) was included in the 8th edition of American Joint Committee on Cancer (AJCC) staging manual for oral cavity cancers^25^. Tumor thickness was used in this study in place of DOI because of the difficulty in retrospective review of tissue sections for DOI. Dirven et al. has reported that tumor thickness can be applied for retrospective survival analyses under incomplete DOI data^26^. The value or significance of pathologic features has not been specifically focused on T3–4 OSCC in previously reported series^6–10,20,22,23^. Anand et al. reported that PNI was an independent adverse risk factor associated with poor locoregional control and survival in stage III-IV bucco-alveolar complex cancers^27^. Jardim et al. also reported PNI to be an independent predictor for overall survival and disease-free survival in clinical stage III-IV oral tongue and floor of mouth cancers^28^. In this study, we focused on T3–4 OSCC and included more oral cavity subsites. These might explain the different independent impacts of PNI in our data. We observed that many clinicopathologic factors correlated with poor survival outcomes in T3–4 OSCC, such as LN+, tumor thickness, PNI, LVI and extranodal extension (ENE) (Table 1), but most factors of primary tumors did not consistently predict LN+, DM and DSS independently. A possible explanation is that the combined influences of coexisting unfavorable factors in larger T3–4 OSCC tumors might result in the loss of independent predictive roles of individual pathologic features. Therefore, the application and significance of pathologic features in T3–4 OSCC is different from T1–2 OSCC and require special considerations.

The concept of combining poor risk factors in head and neck squamous cell carcinoma (HNSCC) has been reported^29^. The rationale of combining PNI and LVI in this study is that they are both pathologic features of the primary tumor that are related to tumor-microenvironmental interactions. Despite being less well understood, PNI has been considered a process of cancer spread distinct from LVI^2^. Both PNI and LVI data are widely available being two required components in pathologic reports of OSCC^11^. The role of PNI in predicting neck metastasis in early T1–2 OSCC has been reported^20,21,30^. We recently reported that the migration ability of OSCC could be enhanced by human peripheral nerves^31^, although the exact mechanisms require further investigation. On the other hand, LVI implies the invasion of tumor cells to blood or lymphatic vessels, a process that has long been accepted as an important step in the tumor metastatic cascade or angiogenesis^32^. Given the distinct metastatic mechanisms of PNI and LVI, our results suggest that combined consideration of PNI and LVI consistently contributes to independent predictive roles for LN+, DM and DSS in T3–4 OSCC.

A lower incidence of DM approximately 10% has been reported for OSCC than for other HNSCC^33,34^. However, our data demonstrated a higher DM rate of 17.3% in an exclusive T3–4 OSCC cohort despite that POCCRT was done at significantly higher percentage among patients who developed DM. Moreover, the 52.9% isolated DM rate was also higher than the reported data^34^. In consistent with previous reports, most DM developed in the lung and occurred early within 2 years after treatment completion in our study. The short mean survival time of 3.76 months after DM diagnosis implies again that DM of OSCC is mostly not salvageable^35,36^. Thus, strategies of prediction and early management of patient high risk for DM before clinical presentation might be important to improve the survival for OSCC. We have reported the value of chest CT especially during follow-up period every 6 months for the first 2 years in high-risk HNSCC patients^37^. Our results thus suggest that PNI/LVI double positive is another high-risk factor indicating the need of chest CT follow-up after treatment completion.

In recent years, DM has become an important type of treatment failure of OSCC with the improved locoregional control^34,38^, and LVI and LN+ have been demonstrated to be an essential marker for DM^32,35,39,40^. By combining PNI and LVI in this study, the 33.3% DM rate in PNI/LVI double positive patients is even higher than 27.3% in LVI-positive, or 31.8% in LN+ patients. Moreover, the impacts of PNI/LVI double positive specifically in the 44 LN+ patients remained consistent despite not statistically significant for DM due to the small patient number (Table 6). The markedly high DM rate of 42.9% and a poor 5-year DSS of 27.7% in LN+, PNI/LVI double positive patients further support the value of combined PNI/LVI in identifying a high-risk subgroup that require additional adjuvant treatment strategies against DM to improve survival.

Current treatment policy for locally advanced OSCC recommend multimodal treatment, including surgery followed by radiotherapy with or without chemotherapy^12^. However, maintenance adjuvant chemotherapy has not been a standard practice for locally advanced OSCC due to unknown benefit and poor tolerance from toxicities. Recently, metronomic chemotherapy, using oral tegafur-uracil, has been reported to improve survival and reduce DM in stage III-IV OSCC treated by surgery or CCRT^41,42^. Cetuximab, a monoclonal antibody targeting epidermal growth factor receptor, has also been shown to be a tolerated and effective regimen for maintenance therapy in recurrent metastatic HNSCC^43,44^. Immune check point inhibitors, such as pembrolizumab for PD-L1 high OSCC, are another type of agents in ongoing clinical trials for maintenance adjuvant therapy in locally advanced HNSCC^45^. For the investigation of the efficacy of these maintenance adjuvant therapies, proper selection of patients at high risk of disease relapse is fundamental in study design.

The major limitation of this study is the relatively small number of T3–4 patients (n = 98) and retrospective study design in our cohort. Another limitation is the use of tumor thickness instead of DOI, which was not routinely reported during the study period. Also, maintenance adjuvant therapy has not been shown to be beneficial in the literature for OSCC^46^. Thus, our results cannot advocate this treatment as a standard for PNI/LVI double positive patients. However, our results suggest that PNI/LVI double positive independently predicts DM in T3-T4 OSCC and can be a widely available selection marker for DM high-risk patients in future maintenance adjuvant therapy studies.

In conclusion, combined consideration of PNI and LVI is important in prognostication and potential clinical application of T3–4 OSCC. PNI/LVI double positive independently predicts LN+, DM, and poor DSS in T3–4 OSCC, and can be used as a good marker to select DM high-risk patients for future novel adjuvant therapy trials with the goal of reducing DM and improving the survival for T3–4 OSCC.

## Materials and methods

### Patient population

From the hospital database, 467 patients with newly diagnosed OSCC who underwent curative surgery at the Department of Otolaryngology- Head and Neck Surgery, Taipei Veterans General Hospital between Jan 2002 and Dec 2010 were reviewed. The patients were staged according to the 7th edition of the AJCC Cancer Staging Manual. Patients with DM at initial diagnosis, head and neck cancer history, radiation history to the head and neck, or synchronous malignancies were excluded. In total, 98 patients with pT3–4 OSCC were included in the present study. All the patients underwent curative surgery, including neck dissection with or without flap reconstruction. Adjuvant therapy was given following institutional treatment guidelines, particularly to patients with multiple neck metastases, close or positive margin, PNI, LVI or ENE. Regular follow-up investigations were arranged, including computerized tomography (CT) scan or magnetic resonance imaging of head and neck area every 3 months for first year, 6 months for the second year, and yearly thereafter. Chest radiograph was done yearly. Chest CT was arranged for high-risk patients, including those suspected for recurrence. The medical records were reviewed and updated, and patients or their families were contacted by telephone if lost to follow-up for more than 1 year. This study was reviewed and approved by the Institutional Review Board (IRB No. 2020-09-005BC) of the Taipei Veterans General Hospital. All methods were performed in accordance with the relevant guidelines and regulations. The IRB waived informed consent for this retrospective study.

### Pathologic features

Pathologic features of the primary OSCC were recorded from the pathology report. The data of DOI was not available during the study period, and tumor thickness was measured from the tumor surface or the ulcer base to the deepest point of invasion. The margins were classified as “positive” or “non-positive.” The “non-positive” margin included dysplasia, close (< 2 mm) or negative margin. A senior pathologist who was blinded to the clinical data re-reviewed all hematoxylineosin stained tissue slides, and special attention was given to PNI and LVI, which were identified throughout the whole area of tumor sections serially under 40× or 100× magnification and were confirmed under 200× magnification. PNI was defined as positive when tumor cell infiltration was identified in any layer of the nerve sheath, or tumor in close proximity involving more than one third of the nerve circumference^2^. LVI was defined as the detection of tumor cell infiltration in endothelial-lined vessels or tumor nests within or attached to the endothelial cell lining of the lymphovascular space. PNI/LVI double positive indicated the presence of both PNI and LVI in the primary OSCC.

### Statistical analysis

The endpoints of this study were to evaluate the association between pathologic features and LN+, local and regional control, DM and survival. Comparisons between categorical data were performed using Pearson’s chi-square test or Fisher’s exact test where appropriate, and the independent effect was analyzed using binary logistic regression models. The survival rates, OS and DSS were analyzed by the Kaplan–Meier method. Cox proportional hazards model was used to test the independent significance. Disease-specific death was defined as death either from the index tumor or from treatment-related events. A *p* value < 0.05 was regarded as statistically significant. All statistical analyses were performed using Statistical Package for Social Sciences software (SPSS v.23 for MAC, SPSS Inc., Chicago, IL).

## References

[1] Bray F (2018). Global cancer statistics 2018: GLOBOCAN estimates of incidence and mortality worldwide for 36 cancers in 185 countries. CA Cancer J. Clin..

[2] Liebig C, Ayala G, Wilks JA, Berger DH, Albo D (2009). Perineural invasion in cancer: A review of the literature. Cancer.

[3] Mohammed RA (2007). Improved methods of detection of lymphovascular invasion demonstrate that it is the predominant method of vascular invasion in breast cancer and has important clinical consequences. Am. J. Surg. Pathol..

[4] Harris EI (2008). Lymphovascular invasion in colorectal cancer: An interobserver variability study. Am. J. Surg. Pathol..

[5] Chatzistefanou I, Lubek J, Markou K, Ord RA (2017). The role of perineural invasion in treatment decisions for oral cancer patients: A review of the literature. J. Craniomaxillofac. Surg..

[6] Jones HB (2009). The impact of lymphovascular invasion on survival in oral carcinoma. Oral Oncol..

[7] Fagan JJ (1998). Perineural invasion in squamous cell carcinoma of the head and neck. Arch. Otolaryngol. Head Neck Surg..

[8] Rahima B, Shingaki S, Nagata M, Saito C (2004). Prognostic significance of perineural invasion in oral and oropharyngeal carcinoma. Oral Surg. Oral Med. Oral Pathol. Oral Radiol. Endod..

[9] Michikawa C (2012). Clinical significance of lymphatic and blood vessel invasion in oral tongue squamous cell carcinomas. Oral Oncol..

[10] Lanzer M, Gander T, Kruse A, Luebbers HT, Reinisch S (2014). Influence of histopathologic factors on pattern of metastasis in squamous cell carcinoma of the head and neck. Laryngoscope.

[11] *College of American Pathologists: Cancer Protocols and Checklists: Lip and Oral Cavity, Version 4.0.0.1* (College of American Pathologists, 2018).

[12] National Comprehensive Cancer Network. Head and Neck Cancers. *Clinical Practice Guidelines in Oncology—v3.* http://www.nccn.org/index.asp (2021).

[13] Langendijk JA (2003). Postoperative radiotherapy in squamous cell carcinoma of the oral cavity: the importance of the overall treatment time. Int. J. Radiat. Oncol. Biol. Phys..

[14] Cooper JS (2004). Postoperative concurrent radiotherapy and chemotherapy for high-risk squamous-cell carcinoma of the head and neck. N. Engl. J. Med..

[15] Bernier J (2004). Postoperative irradiation with or without concomitant chemotherapy for locally advanced head and neck cancer. N. Engl. J. Med..

[16] Shah JP, Gil Z (2009). Current concepts in management of oral cancer–surgery. Oral Oncol..

[17] Haddad RI, Shin DM (2008). Recent advances in head and neck cancer. N. Engl. J. Med..

[18] Aivazian K (2015). Perineural invasion in oral squamous cell carcinoma: Quantitative subcategorisation of perineural invasion and prognostication. J. Surg. Oncol..

[19] Miller ME (2012). A novel classification system for perineural invasion in noncutaneous head and neck squamous cell carcinoma: Histologic subcategories and patient outcomes. Am. J. Otolaryngol..

[20] Tai SK (2012). Treatment for T1–2 oral squamous cell carcinoma with or without perineural invasion: Neck dissection and postoperative adjuvant therapy. Ann. Surg. Oncol..

[21] Tai SK, Li WY, Yang MH, Chu PY, Wang YF (2013). Perineural invasion in T1 oral squamous cell carcinoma indicates the need for aggressive elective neck dissection. Am. J. Surg. Pathol..

[22] Chen TC (2013). The impact of perineural invasion and/or lymphovascular invasion on the survival of early-stage oral squamous cell carcinoma patients. Ann. Surg. Oncol..

[23] Liao CT (2012). Identification of a high-risk group among patients with oral cavity squamous cell carcinoma and pT1-2N0 disease. Int. J. Radiat. Oncol. Biol. Phys..

[24] Rajappa SK (2019). Oncological benefits of postoperative radiotherapy in node-negative early stage cancer of the oral cavity with isolated perineural invasion. Br. J. Oral Maxillofac. Surg..

[25] Amin MB, Edge SB, Greene FL (2017). AJCC Cancer Staging Manual.

[26] Dirven R (2017). Tumor thickness versus depth of invasion—Analysis of the 8th edition American Joint Committee on Cancer Staging for oral cancer. Oral Oncol..

[27] Anand AK (2017). Significance of perineural invasion in locally advanced bucco alveolar complex carcinomas treated with surgery and postoperative radiation ± concurrent chemotherapy. Head Neck.

[28] Jardim JF, Francisco AL, Gondak R, Damascena A, Kowalski LP (2015). Prognostic impact of perineural invasion and lymphovascular invasion in advanced stage oral squamous cell carcinoma. Int. J. Oral Maxillofac. Surg..

[29] Lin YT (2015). Triple-positive pathologic findings in oral cavity cancer are related to a dismal prognosis. Laryngoscope.

[30] Chatzistefanou I, Lubek J, Markou K, Ord RA (2014). The role of neck dissection and postoperative adjuvant radiotherapy in cN0 patients with PNI-positive squamous cell carcinoma of the oral cavity. Oral Oncol..

[31] Lee TL (2019). Nerve-tumour interaction enhances the aggressiveness of oral squamous cell carcinoma. Clin. Otolaryngol..

[32] Guo W, Giancotti FG (2004). Integrin signalling during tumour progression. Nat. Rev. Mol. Cell Biol..

[33] Uchiyama Y (2020). Distant metastasis from oral cavity-correlation between histopathology results and primary site. Oral Radiol..

[34] Sumioka S (2013). Risk factors for distant metastasis in squamous cell carcinoma of the oral cavity. J. Oral Maxillofac. Surg..

[35] Barros-Silva PG (2020). Clinical-pathological and sociodemographic factors associated with the distant metastasis and overall survival of oral cavity and oropharynx squamous cell carcinoma. Med. Oral Patol. Oral Cir. Bucal.

[36] Sakamoto Y (2016). Risk factors of distant metastasis in patients with squamous cell carcinoma of the oral cavity. Oral Surg. Oral Med. Oral Pathol. Oral Radiol..

[37] Hsu YB (2008). Role of chest computed tomography in head and neck cancer. Arch. Otolaryngol. Head Neck Surg..

[38] Cracchiolo JR (2018). Patterns of recurrence in oral tongue cancer with perineural invasion. Head Neck.

[39] Aires FT, Lin CS, Matos LL, Kulcsar MAV, Cernea CR (2017). Risk factors for distant metastasis in patients with oral cavity squamous cell carcinoma undergoing surgical treatment. ORL J. Otorhinolaryngol. Relat. Spec..

[40] Bugshan A, Farooq I (2020). Oral squamous cell carcinoma: metastasis, potentially associated malignant disorders, etiology and recent advancements in diagnosis. F100Res.

[41] Huang WY, Ho CL, Chao TY, Lee JC, Chen JH (2021). Oral tegafur-uracil as a metronomic therapy in stage IVa and IVb cancer of the oral cavity. Am. J. Otolaryngol..

[42] Hsieh MY, Chen G, Chang DC, Chien SY, Chen MK (2018). The impact of metronomic adjuvant chemotherapy in patients with advanced oral cancer. Ann. Surg. Oncol..

[43] Addeo R (2018). Maintenance therapy with biweekly cetuximab: Optimizing schedule can preserve activity and improves compliance in advanced head and neck cancer. Oncology.

[44] Vermorken JB (2008). Platinum-based chemotherapy plus cetuximab in head and neck cancer. N. Engl. J. Med..

[45] Machiels JP (2020). Pembrolizumab given concomitantly with chemoradiation and as maintenance therapy for locally advanced head and neck squamous cell carcinoma: KEYNOTE-412. Future Oncol..

[46] Pignon JP, le Maitre A, Maillard E, Bourhis J (2009). Meta-analysis of chemotherapy in head and neck cancer (MACH-NC): An update on 93 randomised trials and 17,346 patients. Radiother Oncol..

